# Detection and discrimination of flicker contrast in migraine

**DOI:** 10.1177/0333102411398401

**Published:** 2011-04

**Authors:** Olivera Karanovic, Michel Thabet, Hugh R Wilson, Frances Wilkinson

**Affiliations:** York University, Canada.

**Keywords:** Migraine, vision, flicker, contrast threshold, adaptation, habituation

## Abstract

*Aims:* Flickering light is strongly aversive to many individuals with migraine. This study was designed to evaluate other abnormalities in the processing of temporally modulating visual stimulation.

*Methods:* We measured psychophysical thresholds for detection of a flickering target and for the discrimination of suprathreshold flicker contrasts (increment thresholds) in 14 migraineurs and 14 healthy controls with and without prior adaptation to high-contrast flicker. Visual discomfort (aversion) thresholds were also assessed.

*Results:* In the baseline (no adaptation) conditions, detection and discrimination thresholds did not differ significantly between groups. Following adaptation, flicker detection thresholds were elevated equivalently in both groups; however, discrimination thresholds were more strongly affected in migraineurs than in controls, showing greater elevation at moderate contrasts and greater threshold reduction (sensitisation) at high contrast (70%). Migraineurs also had significantly elevated discomfort scores, and these were significantly correlated with number of years with migraine.

*Discussion:* We conclude that visual flicker not only causes discomfort but also exerts measurable effects on contrast processing in the visual pathways in migraine. The findings are discussed in the context of the existing literature on habituation, adaptation and contrast-gain control.

## Introduction

Photophobia or aversion to light is one of the defining characteristics of migraine headache (1). Although this is most intense during the headache phase, many migraineurs report heightened visual sensitivity between migraine episodes (2,3), and as a prodromal symptom as well (4). In addition to finding normal levels of illumination too bright or even painful, specific spatial and temporal patterning of light have been reported to be particularly aversive. Controlled investigations of this phenomenon have focused largely on its spatial properties. Spatially redundant patterns (high-contrast gratings) have been reported to elicit feelings of “visual discomfort” and to give rise to a range of visual illusions, including motion, orientation distortion and colour (2,3,5,6). The term *pattern glare* has been coined for these spatial phenomena, and interictal pattern glare has been reported to be correlated with performance on certain other visual tasks (3,7).

Although it has been less studied, temporal repetition or flicker is generally far more aversive to migraineurs than spatial redundancy. Anecdotally, migraineurs report being bothered by television and computer screens, flickering fluorescent lights and flickering sunlight when driving past avenues of trees (8). In the laboratory, during a task in which the contrast of a flickering screen is increased until it becomes aversive, migraineurs (both with [MA] and without [MO] visual aura) aborted the test at significantly lower contrasts than individuals with no migraine history, at all temporal frequencies tested (1–30 Hz inclusive) (9); greatest sensitivity was seen at and above 10 Hz. In a recent study of binocular rivalry, we also monitored aversion to a 10-Hz flickering stimulus, and found high sensitivity in most migraineurs but in few control participants (10); weaker effects were seen for static grating patterns. The percept evaluated in all of these studies was aversiveness. Although it seems probable that this would be related to other measures of sensitivity to spatiotemporal variation, such as threshold contrast sensitivity and suprathreshold discrimination ability, the link has not been directly examined for temporal sensitivity; Shepherd has provided evidence of such a link for spatial contrast (7).

Dynamic stimuli (flickering, phase reversing or drifting patterns) have been used in several studies of contrast detection thresholds in migraine. Benedek et al. (11) reported sensitivity loss in MO for low spatial frequency gratings, both static and dynamic (phase reversing at 1 Hz), which they attributed to a magnocellular pathway deficit. In a group of MA subjects, McKendrick et al. (12) reported normal sensitivity to a low-spatial-frequency pattern drifting at 16 Hz both foveally and 10° into the periphery. Khalil evaluated both spatial and temporal (10–30 Hz) contrast sensitivity in migraine and found elevated thresholds in MA and not in MO (13). This is the only study to systematically examine sensitivity to high temporal frequencies (10–30 Hz). In a series of studies using the Medmont perimeter, McKendrick and colleagues have reported areas of reduced sensitivity to flicker at contrast threshold, predominantly in the peripheral visual field (14–16). However, frequency-doubling perimetry, which is also based on temporal contrast sensitivity, has generally failed to reveal impairments in migraine (17,18). Aversiveness was not assessed in any of these threshold studies.

Flicker discrimination at higher contrasts has not been studied psychophysically in migraine. However, electrophysiological responses to high-contrast flickering stimuli with diverse characteristics have been studied extensively using visual evoked potentials (VEPs) in migraineurs [reviewed in (19,20)]. While there are some inconsistencies in the literature, the most common report is of higher amplitude visual evoked responses in migraineurs than in controls. With repeated stimulation, the amplitude of the normal VEP declines gradually (habituation), whereas in migraineurs, VEP amplitude either remains unchanged or may even increase (potentiation) (21). Similar effects have been reported in other sensory modalities (22–26). Coppola et al. (27) have summarised this growing literature and argue that there is a generalized abnormality in habituation in the migraine nervous system. For reasons to be addressed in the discussion, we will use the term “adaptation” rather than “habituation” to describe exposure to a prolonged visual stimulus. However, the observation that the response of the nervous system is affected differently by prolonged or repetitive stimulation in migraine has important implications for understanding the neural basis of this disorder. In many ways migraine appears to be a disorder of “sensory gain”. During migraine episodes, normal levels of stimulation in all the sensory modalities give rise to extreme discomfort or pain (photophobia, phonophobia, allodynia, osmophobia), percepts normally elicited only at the highest amplitudes of stimulation.

In order to more clearly elucidate the role of flickering visual stimuli in migraine, the present study was designed with the following two goals. The first was to assess sensitivity to small differences in the contrast of pairs of very brief flickering stimuli (increment thresholds). In the contrast detection test, the minimum detectable contrast increment above the background (0% contrast) luminance level was measured. In the contrast discrimination tasks, the smallest detectable increments above two base (or pedestal) contrasts (10% and 70%) were measured. The second goal of the study was to assess the effects of adaptation on these contrast increment thresholds, looking for a psychophysical analogue to the electrophysiological habituation results reported in the literature.

As described at the outset, this work is motivated by the observed aversiveness and, in some cases, headache-inducing properties (28) of flickering stimuli. This posed a serious problem in designing the study. To obtain good psychometric functions requires extensive testing of trained subjects. However, migraineurs face a risk of bringing on a headache as a result of testing, especially in the habituation paradigm, if testing is prolonged. Therefore, we focused our study on a group of migraineurs who are working or studying at the York University campus and who were able to come to the laboratory for multiple, relatively brief sessions over a period of several weeks. Because our earlier work has found that both MA and MO groups show very significant sensitivity to flicker (9,10), we did not attempt to recruit separate MA and MO groups.

## Methods

### Participants

The study was approved by the York University Human Participants Review Committee (the University’s research ethics committee). Subjects were recruited by advertisements posted on the York University campus. All potential participants underwent an extensive, structured clinical interview including a detailed description of their headache history. Questions were designed in accordance with current International Headache Society (1) diagnostic criteria, allowing classification of their headaches. The questionnaire also evaluated exclusionary criteria applied to all groups, including cardiovascular disease, diabetes, neurological disorders (epilepsy, optic neuritis, multiple sclerosis) and history of substance abuse or severe head or neck injury. Subjects were also excluded if they were suffering from any visual abnormality not corrected by optical lenses (e.g. amblyopia). An additional basis for exclusion was treatment with any prophylactic medication for migraine or mood disorders. Control participants with a positive family history of migraine or more than one tension headache per month were excluded from the study. Written informed consent was obtained from all participants, and in addition, migraineurs consented to confirmation of their migraine status by their primary care physician whenever possible. Monocular and binocular acuities and contrast sensitivity were measured using Stimuli™ version 3.5 (Haag-Streit, Mason, OH, USA) calibrated for a distance of 3.66 m. Stereopsis was measured with the Titmus test (Stereo Optical, Chicago, IL, USA).

### Stimuli

Visual stimuli were generated on a Macintosh G4 computer using VPixx™ software (VPixx Technologies, St. Bruno, QC, Canada) and presented on a 19-inch Samsung CRT monitor at a resolution of 800 x 600 pixels and a frame rate of 120 Hz. The mean luminance of the screen was 54 cd/m^2^.

The test and adapting stimuli were flickering spots with blurred edges (Gaussian luminance profiles, σ = 0.5° and 1°, respectively). The approximate visible diameter of the test stimuli was 1°; the adapting stimuli were twice this diameter, and all stimuli were centered at fixation. The larger adapting spots ensured that small involuntary eye movements during adaptation did not reduce the level of adaptation at the fixation point, where the test spots were presented. Both test and adapting spots underwent square-wave flicker at a temporal frequency of 10 Hz around the background screen luminance.

Based on extensive pilot testing at a wider range of contrasts in a small subgroup of participants (four healthy controls [HCs] and three migraineurs, as described below in “Results”, base contrasts of 0% (absolute contrast threshold), 10% and 70% were chosen as most likely to reveal the effects of flicker adaptation and differences between migraineurs and controls. Contrast for a Gaussian spot is defined as the difference between the peak luminance of the Gaussian and the mean luminance divided by the mean luminance.

## Procedure

Participants were seated in a chair positioned 1.14 m from the computer screen under dim room illumination. Head position and distance from the screen were controlled using a chin and forehead rest. During the experiment participants wore their corrective lenses if applicable. All testing was conducted binocularly and stimuli were presented foveally.

**Figure 1.**
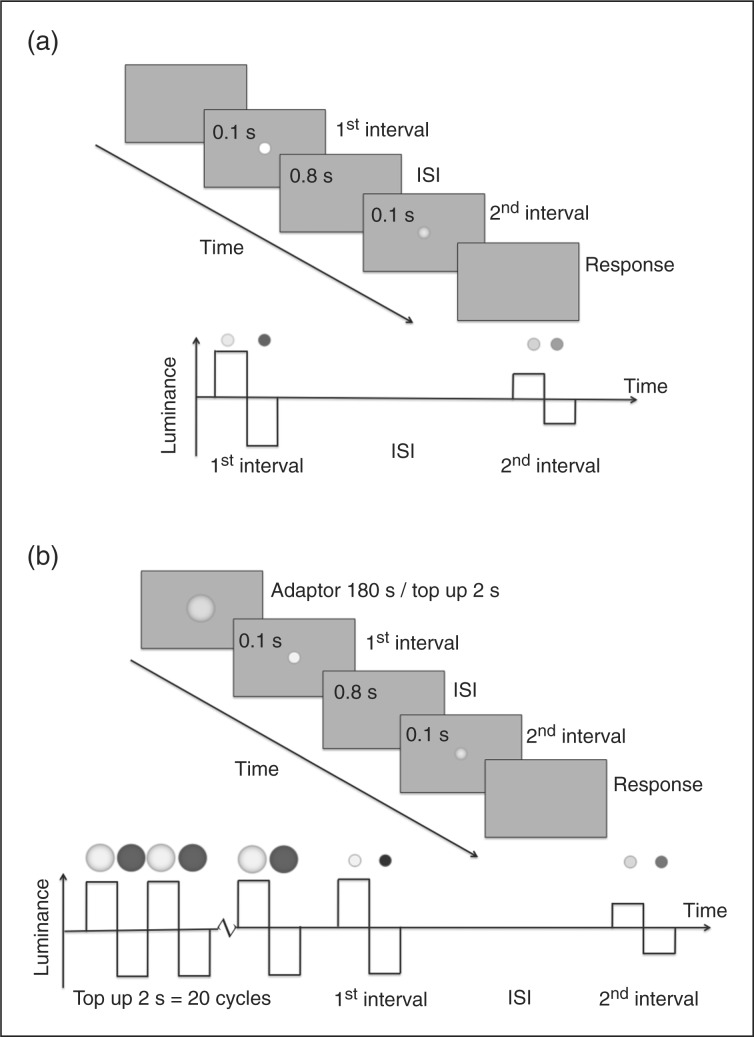
Time line for a single trial of the experiment. (a) Baseline condition. Each test interval consists of one cycle of 10-Hz square-wave flicker. The stimuli were always in the same phase (luminance increment followed by luminance decrement. (b) Adaptation condition. Three minutes of adaptation preceded the first trial and every subsequent trial was preceded by two seconds of top-up flicker adaptation. In the detection task, because only the increment target would be visible, the target location was cued by four small lines surrounding the target location (eccentricity 1.25°). This fixation cue appeared for 0.2 seconds before each interval. s = seconds. ISI = inter-stimulus interval.

*Baseline conditions:* Contrast detection and discrimination thresholds were measured in a two-interval forced choice paradigm. The two stimuli each consisted of a single cycle of flicker (0.1 seconds) and were separated by an inter-stimulus interval (ISI) of 0.8 seconds (Figure 1a). The order of presentation of the base and increment stimuli was randomized across trials, and the subject’s task was to indicate by a key press whether the higher-contrast stimulus occurred in the first or the second interval. In the contrast detection task (0% base contrast), no target was presented in one interval and a low-contrast target in the other interval; in this condition only, the location of the targets was indicated by four small flanking bars. In the contrast discrimination tasks, the base stimulus (10% or 70% contrast) appeared in one interval and a small increment above this level in the other interval. Each test run began at a highly visible stimulus increment and thresholds were tracked using a 3-down, 1-up staircase (contrast was reduced after three correct responses and increased after a single error) yielding a 79% correct threshold level. The last eight out of 12 reversal points were included in the calculation of threshold.

*Adaptation conditions* (Figure 1b): The adaptor was a Gaussian spot of double the diameter of the test spot, presented at fixation at 70% contrast with a flicker rate of 10 Hz. At the beginning of each adaptation experiment, the adaptor was presented for 180 seconds and before each test trial the adaptor was presented for 2 seconds to top up adaptation. In all other respects, baseline and adaptation threshold measurements were identical.

All subjects went through an intensive, step-by-step training process to ensure that they were familiar and comfortable with the task. Participants were asked to fixate at the centre of the screen, and to initiate each run by pressing the space bar on the keyboard. The targets were briefly presented in a sequence and participants had to indicate by key press (1 or 2) whether the first or second stimulus was of higher contrast. During the training all contrast conditions were shown and feedback was provided. All subjects then completed four threshold measurements at each contrast level under baseline and adaptation conditions, 24 thresholds in total. Blocks of baseline (B) and adaptation (A) threshold measurements at the three-base contrast levels were alternated in ascending (B0%, B10%, B70%; A0%, A10%, A70%) and then descending order (B70%, B10%, B0%; A70%, A10%, A0%). This was repeated until all threshold measurements were complete. Within a session, a baseline block was always performed before adaptation. Participants were encouraged to take breaks between runs.

Our standard visual discomfort test for flicker (10) was administered to all subjects at the end of their last session. The test began with a full-field gray screen (18.1° x 13° at the viewing distance of 1.14 m; mean luminance of 54 cd/m^2^). Luminance was then temporally modulated at 10 Hz as contrast was increased in equal steps every five seconds until the pattern became aversive and the test was aborted by the participant, or until 100% contrast was reached. The procedure was repeated five times.

Each participant took approximately five hours, split into five to six sessions, to complete all 24 increment threshold runs together with the training and the flicker discomfort measure. All participants had been headache-free for at least four days at the time of each testing session.

## Data analysis

Contrast increment thresholds have been shown to have a power-law relationship to base contrast over a considerable portion of the contrast range, meaning that this relationship is a straight line when contrast and increment threshold are plotted on log-log coordinates (29). Therefore, all data analysis was performed on log-transformed threshold measures. Threshold estimates from the four tests at each contrast/adaptation condition were averaged to yield a single mean value for each participant at each level. Threshold elevation scores were also calculated for each participant at each contrast level by taking the difference between the log-transformed adapted and baseline thresholds. This provides a measure of the effect of adaptation independent of individual differences in unadapted baseline thresholds.

Scores on the visual discomfort flicker test (highest contrast reached before test was aborted) were averaged for each participant across the five trials; these values were then converted to discomfort scores by applying the formula: *discomfort threshold = log (1/average contrast score)*. This yields a discomfort scale on which 0 indicates no discomfort even at the highest possible contrast, 1 represents a discomfort threshold at 10% contrast and 2 represents a threshold of 1% contrast (10).

All data sets were evaluated for departures from normality using the Kolmogorov-Smirnov test, and for homogeneity of variance using Levene’s F. Where appropriate, parametric statistics (split-plot analysis of variance [ANOVA] or analysis of co-variance [ANCOVA]) were then applied to the data. In cases where the assumptions of parametric statistics were not met, non-parametric tests (Mann-Whitney U [for unpaired data], or Wilcoxon [for paired data]) were used.

Correlations were assessed between all threshold data sets, discomfort scores and each of the following subject factors: age, migraine duration (years with migraine), and migraine frequency (episodes/year). Spearman’s rank correlation was used when the distribution of a subject variable was strongly skewed.

## Results

### Participants

The study sample consisted of 14 migraineurs (nine MO, five MA; mean age in years 23.93 ± 6.84); and 14 age- and gender-matched migraine-free control participants (12 female and 2 male, mean age in years 28.71 ± 9.19). All participants had normal stereopsis, normal or corrected to normal visual acuity (20/25 or better) and contrast sensitivity (2.4% or better) (see Table 1 for participant details). The distribution of refractive errors (REs in Table 1) and interocular differences in refractive errors (before correction) were very similar for the two groups, and were not significantly correlated with any of the threshold measurements or the migraine characteristics (duration, severity) as assessed by Spearman’s ρ (*p* > .05 in all cases). Only one participant in each group had refractive errors >3.75D. All participants wore appropriate correction during testing.

**Table 1.** Group characteristics

	HCs (*N* = 14)	Migs (*N* = 14)	MO (*N* = 9)	MA (*N* = 5)
Age: mean ± SD (range)	28.7 ± 9.19 (19–52)	23.9 ± 6.84 (18–43)	23.8 ± 7.68 (18–43)	24.2 ± 5.85 (20–34)
Ratio of women to men	12:2	12:2	7:2	5:0
Years with migraine: mean ± SD	N/A	10.71 ± 8.51	9.89 ± 10.39	12.2 ± 3.9
Migraine headache frequency/month: mean ± SD	N/A	1.81 ± 2.02	2.5 ± 2.26	0.57 ± 0.18
Aura frequency/year mean ± SD in MA	N/A	4.0 ± 2.51	N/A	4.0 ± 2.51
Binocular acuity median (range)	20/15 (20/10– 20/20)	20/15 (20/10– 20/20)	20/15 (20/10– 20/20)	20/15 (20/10– 20/15)
Binocular contrast sensitivity median (range)	1.2 % (1.2–2.4)	1.2% (0.6–1.2)	1.2% (0.6–1.2)	1.2% (0.6–1.2)
REs before correction (worst eye) median (range)	0.75D (0–6.25)	0.62D (0–6.75)	0.75D (0–6.75)	0D (0–1.75)
Interocular difference in REs before correction median (range)	0D (0–1.5)	0.13D (0–1.75)	0.25D (0–1.25)	0D (0–0.25)

HCs = healthy controls. Migs = migraineurs. MO = migraine without aura. MA = migraine with aura. SD = standard deviation. N/A = not applicable. REs = refraction errors.

Twelve additional participants (nine migraineurs, three HCs) withdrew before completion of the study. Three migraineurs experienced frequent migraine headaches possibly induced by the task, and two migraineurs started antidepressant/pain medications. Two (one Mig, one HC) found the task too difficult, and the others withdrew because of time constraints (three migraineurs, one HC) or for other, undisclosed reasons (one HC). The very demanding nature of the task, both in terms of time and concentration required, and in the case of migraineurs, the aversiveness of the stimuli, account for the unusually high drop-out rate.

### Age

Migraine participants were on average somewhat younger than controls (23.9 ± 6.8 versus 28.7 ± 9.2 years); this difference was not statistically significant (t_24_ = 1.56; *p* = .13). Correlation between increment thresholds and age for control subjects did not reach statistical significance in any test condition; however, all correlations were positive (range: r = 0.23–0.36) suggesting that age might contribute to elevating thresholds in general. For this reason, and because of the difficulty of recruiting and retaining migraine participants, making precise age-matching difficult, age was included as a covariate in the analysis of increment thresholds. Because age is generally highly correlated with years with migraine, any cumulative effects of migraine might either enhance or act against an underlying age effect.

### Migraine with versus without aura

Within the migraine group, those with MA and MO were closely comparable in age (mean 23.8 vs*.* 24.2 years); individuals with MA had somewhat longer migraine history (12.2 vs. 9.9 years) but less frequent episodes (0.57 vs. 2.5/month). Only the frequency difference reached statistical significance (t = 2.5; *p* = .03). The performance of MA and MO participants was comparable in range and in pattern so they are presented as a single group in the following analyses.

### Contrast increment thresholds

**Figure 2.**
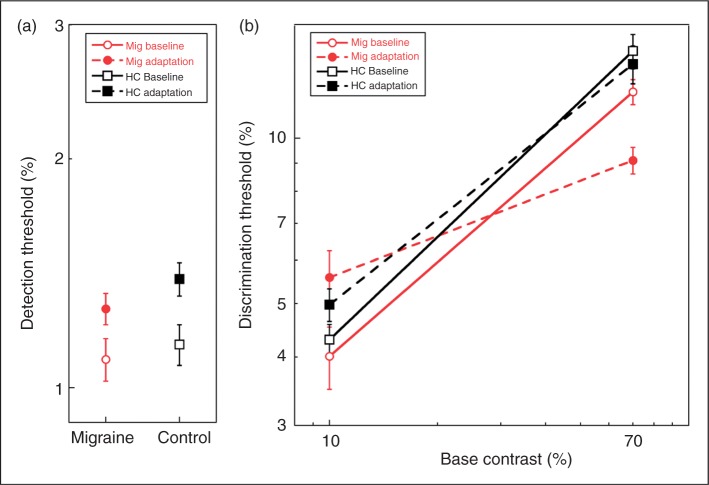
Contrast increment thresholds for migraine and healthy control participants. Open symbols represent unadapted baseline thresholds; filled symbols, thresholds following adaptation to 70% contrast 10-Hz flicker. (a) Detection thresholds for migraineurs and healthy control participants. (b) Discrimination thresholds tested at 10% and 70% base contrast. Note that the Y axes in 2a and 2b cover different ranges; all Y-values in 2a lie below any value in 2b. Error bars = ±1 standard error. Mig = migraineurs. HC = healthy controls.

The baseline contrast thresholds and the thresholds following adaptation to 10-Hz flicker are shown for contrast detection (0%) in Figure 2a, and for contrast discrimination (10% and 70%) in Figure 2b. Increment thresholds increase with base contrast over the range of base contrasts tested here. The effects of migraine condition and adaptation are complex, as they differ across contrasts. Generally, adaptation elevates thresholds at low contrasts and depresses them at high contrast; however, the strength of this pattern differs for Mig and HC groups.

The log transformed data set did not violate the assumptions of either normality (Kolmogorov-Smirnov test: *p* > .05 in all cases) or homogeneity of variance (Levene’s F; *p* > .05 in all cases). Because there was a slight, although not statistically significant, difference in the age of the two groups (see above), age was entered as a covariate in the following analysis. The threshold data were submitted to split-plot ANCOVA with migraine status (migraine, control) as the between group factor, age as covariate and contrast level and adaptation condition (adaptation, baseline) as the within-group factors. Sphericity was not violated in this analysis. The results of the split-plot ANCOVA revealed a significant main effect of contrast (F_2,50_ = 71.93; *p* < .001), and a significant three-way group x adaptation x contrast interaction (F_2,50_ = 9.06; *p* < .001). No other main effects or interactions reached statistical significance (*p* > .05).

In order to interpret this complex interaction effect, we examined simple effects at each base contrast. At each contrast level, we evaluated the effect of adaptation condition on each group, and the effect of group on each adaptation level, using Bonferroni’s correction for four comparisons at each level (adjusted significance level: *p* < .0125). At absolute threshold, adaptation elevated thresholds significantly over their baseline level in both migraineurs (F_1,26_ = 12.78; *p* < .01) and HCs (F_1,26_ = 18.48; *p* < .001). Mig and HC groups did not differ from each other under either adaptation (F_1,26_ = 0.73; *p* = .40) or baseline conditions (F_1,26_ = 0.079; *p* = .78). At 10% base contrast, adaptation significantly elevated thresholds in migraineurs (F = 27.3; *p* < .0001); the same trend was evident in the HC group but did not reach statistical significance (F = 2.84; *p* = .10). At this contrast, the groups did not differ from one another under either adaptation condition (baseline: F_1,26_ = 1.14; *p* = .29; adaptation: F_1,26_ = 0.88; *p* = .36). The picture is different at 70% contrast. Here the effect of adaptation was to significantly reduce thresholds in the Mig group (F_1,26_ = 21.71; *p* < .001). While the trend is in the same direction for HCs, the adaptation and baseline thresholds were not significantly different for this group (F_1,26_ = 1.31; *p* = .26). Without adaptation, Mig and HC increment thresholds did not differ (F_1,26_ = 2.19; *p* = .15), whereas under high contrast adaptation, increment thresholds were significantly reduced in migraineurs compared to HC (F_1,26_ = 12.70; *p* = .001).

The main effect of base contrast is evident in the Figure 2a and b. Detection thresholds were lower than thresholds at 10% base contrast (*p* < .001), which were in turn lower than at 70% contrast (*p* < .001). This was true in migraineurs and HCs for both baseline and adaptation.

### Threshold elevation

**Figure 3.**
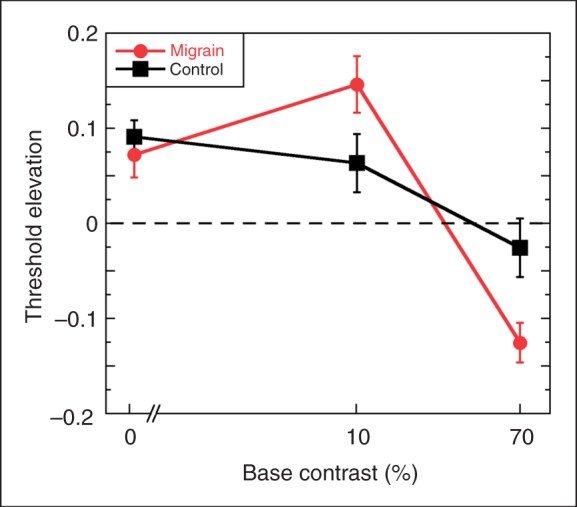
Threshold elevation ratios calculated as the difference between the log thresholds for the adapted and baseline conditions. Positive values indicate that thresholds were elevated following adaptation, negative values indicate improved sensitivity following adaptation and 0 indicates no change. Error bars denote ± 1 standard error.

The effect of adaptation, scaled to non-adaptation baselines, was examined using ratio scores (difference of logs) calculated for each subject (Figure 3). In this figure, positive values indicate that adaptation elevated thresholds, whereas negative scores indicate a reduction in threshold (or increased sensitivity) following adaptation. A score of 0 would indicate that adaptation had no effect on threshold. Comparing each of these ratios to 0 using t-tests confirmed the simple effects reported earlier: while migraineurs showed a significant effect of adaptation at all contrasts (*p* < .01 in all cases), only the threshold elevation at detection threshold (or 0% contrast) was statistically significant for the HC group. Split-plot ANOVA revealed a highly significant main effect of contrast (F_2,52_ = 37.19; *p* < .0001) and interaction between group and contrast (F_2,52_ = 8.07; *p* < .001); the main effect of group was not significant (F_1,26_ = 0.22; *p* = .64). As previously noted, adaptation elevated thresholds at 0% and 10% contrast. The size of the adaptation effect did not differ between groups at detection threshold (F_1,26_ = 0.26; *p* = .60). At 10% contrast, migraineurs showed a marginally greater threshold elevation than HC (F_1,26_ = 3.40; *p* = .064), whereas at 70% contrast, migraineurs showed a significantly greater depression in thresholds than HC (F_1,26_ = 11.62; *p* < .01).

### Slope of the contrast function: individual data

We examined the individual scores for 10% and 70% contrast in the Mig group and found that the crossover effect illustrated in Figures 2b was seen in 11 of the 14 migraineurs. The remaining three showed similar slope differences, but the curves did not cross over, two meeting at 10% and one at 70%, suggesting that it is only the point of crossover and not the pattern that differed in these participants. The situation in the HC group was quite different. Six of 14 HCs showed a similar crossover pattern to the Mig group, although the difference in slopes was smaller. The remaining eight control subjects showed a different pattern—in all cases, adapted performance was parallel to unadapted baseline, but in five cases adaptation elevated thresholds and in the remaining three cases it reduced thresholds.

**Figure 4.**
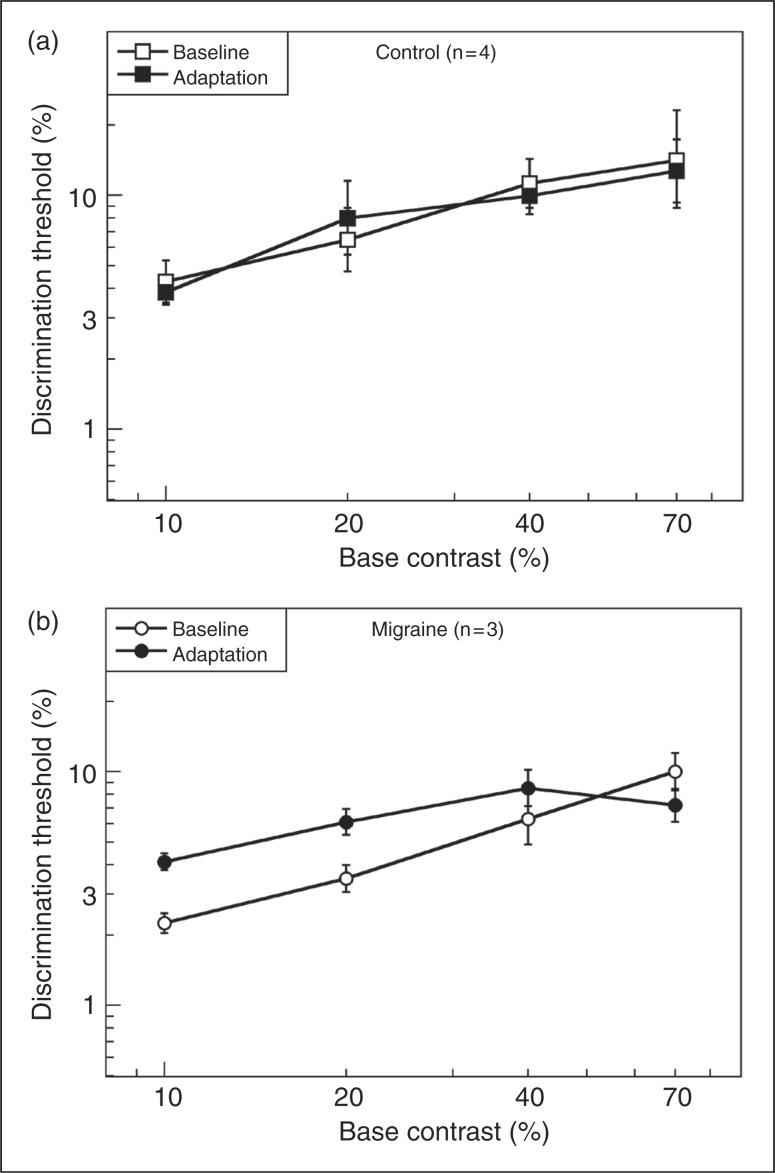
Discrimination thresholds for (a) four healthy control participants tested at 4 base contrasts, and (b) three migraine participants tested at the same base contrasts. Error bars denote ± 1 standard error.

Two data points do not provide an adequate basis for describing the shape of a function, so it was important to obtain information about increment thresholds for contrasts between 10% and 70%. We were able to obtain measurements at 20% and 40% contrast, in addition to 10% and 70% in seven individuals, four controls and two migraineurs from the main study and one additional MA subject who was tested in pilot work but was not included in the main study group as she lay outside the age range of the other participants (60 years). Results for these two groups, averaged across four and three individuals respectively are shown in Figures 4a and b. It is clear that the controls show very little effect of adaptation at any base contrast (except at detection threshold; not shown). However, all three migraineurs showed the effect described earlier for the larger group; the pattern of crossover shown in Figure 4b was identical in each of these participants, occurring between 40% and 70% base contrast in every case.

### Visual discomfort

**Figure 5.**
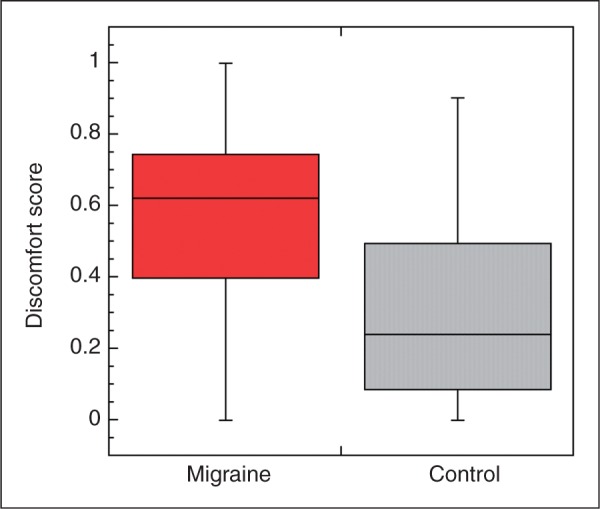
Discomfort scores for full-field flickering screen for migraine and healthy control groups. Box plots show median (solid line), 25 and 75% quartiles (limits of boxes) and range of the data sets. Discomfort score of 0 indicates that flicker was tolerated even at maximum (100%) contrast; higher scores indicate lower thresholds for aborting the stimulus.

The discomfort scores for Mig and HC groups are shown in box and whisker plots in Figure 5. The Mig group was significantly more sensitive to flicker than the HC group, aborting the stimulus at a lower median contrast level (Mann-Whitney test: *p* = .038). It should be noted, however, that two migraineurs experienced little or no discomfort at any contrast. Discomfort scores were not significantly correlated with detection thresholds, discrimination thresholds or threshold elevation scores in either group (Spearman’s ρ, *p* > .05 in all cases).

### Correlations with subject variables

Correlations between migraine variables (duration: years with migraine; frequency: episodes per month) and all experimental measures were evaluated with Spearman’s correlation for ranks. Visual discomfort scores were strongly positively correlated with migraine duration (ρ = 0.83, *p* < .001) but not with migraine frequency (ρ = −0.30; *p* = .29). Turning to the increment threshold findings, there were no significant correlations between baseline flicker detection thresholds (0% base contrast), post-adaptation detection thresholds or threshold elevations and any of the migraine variables. At 10% base contrast, migraine duration was significantly positively correlated with post-adaptation threshold (ρ = 0.62; *p* = .019) and with threshold elevation ratio (ρ = 6; *p* = .023) but not with baseline threshold; participants with longer migraine histories showed larger elevations in threshold following adaptation. On the other hand, at 70% contrast, threshold elevation was significantly negatively correlated with migraine frequency (ρ = −0.59; *p* = .03). This negative correlation reflects the threshold reduction induced by adaptation at high contrast in migraine, as illustrated in Figure 3. It must be emphasised that we have not applied a correction for multiple tests to the reporting of these correlations and the significance levels are quite marginal, so establishing the validity of these correlations will depend on replication in larger samples of migraineurs.

## Discussion

The present findings again confirm the sensitivity of migraineurs to flickering light. The discomfort scores recorded in this study replicate our earlier findings using the same measure (10); the median scores in the two studies are nearly identical although only one individual participated in both studies. This provides quantitative support for the common anecdotal reports from migraineurs that they are bothered by a wide range of flickering patterns from fluorescent lights and television screens to sunlight viewed through trees from a moving automobile. However, the discomfort scores speak only to the aversive quality of this stimulus and not to whether the visual pathways respond differently to flicker in other respects. This is addressed by the contrast detection and discrimination threshold data.

These findings may be summarized in three points. (i) Detection thresholds (0% base contrast) did not differ between migraineurs and HCs, and the groups showed equivalent elevations in contrast detection thresholds following adaptation to high-contrast flicker. (ii) Baseline discrimination thresholds did not differ significantly between migraineurs and controls; there was a slight trend toward lower thresholds in migraineurs at both contrasts tested. (iii) Adaptation to 70%-contrast flicker significantly elevated contrast discrimination thresholds at moderate contrast (10%) and significantly lowered discrimination thresholds at high contrast (70%) in migraineurs. Very weak trends in the same direction were evident in the control group but their results did not differ statistically from the unadapted baseline condition at either contrast. Thus, overall, adaptation exerts a much stronger effect on the visual system of migraineurs than on that of controls. We address each of these points below*.*

### Contrast detection thresholds

The present result of normal contrast detection thresholds for flicker in migraine are consistent with the findings of Khalil (13) in MO subjects across a wide range of temporal frequencies, including the 10 Hz used here; however, he reported elevated thresholds in MA. While only five of the 14 participants in the present study were MA, we saw no evidence in terms of mean, median or range of scores of any difference in this direction. An important difference between our methodology and Khalil’s was that he used the method of adjustment so the stimulus was continuously flickering and the subject indicated when the flicker reached a perceptible contrast, whereas in the present case we used individual presentations of a single flicker cycle of the stimulus. Our results are also consistent with McKendrick’s report of normal thresholds for foveal detection of a Gabor pattern drifting at 16 Hz in a group of MA subjects.

At first sight, our results seem in conflict with a number of other findings from McKendrick and collegues. Flicker perimetry studies (14–16,30) have revealed both global and localized measures of reduced sensitivity in migraine subjects, particularly in the peripheral fields. In studies by the same group using a pair of measures which isolate magnocellular (M) from parvocellular (P) sensitivity (31), migraineurs were reported to show elevated thresholds (reduced sensitivity) to both stimuli in the periphery (implicating both M and P pathways) but normal performance in the fovea (30,32). One possible explanation for the discrepancy is that the present study used foveal stimuli only. It will be important in future studies to extend our paradigm to the peripheral visual field. Another difference is that both the Medmont flicker perimetry task and the Pokorny/Smith task (31) involve a step in local mean luminance as well as a transient stimulus, which is known to affect flicker thresholds in normal vision (33). Thus, the problem for migraineurs may not be in flicker or step detection per se, but in achieving the rapid local-light adaptation change necessary for optimal detection performance. The present study does not address the temporal dynamics of visual adaptation.

The present findings of marked elevations in detection threshold following adaptation to flicker are consistent with reports in the literature on normal vision; prolonged high contrast flicker exposure has been shown to affect thresholds for flicker (34–36) and for other magnocellular-selective stimuli, such as global motion coherence (37). In the present study, control and migraine participants showed similar threshold elevations (Figure 3), suggesting that the mechanism through which adaptation affects detection threshold is not selectively affected by migraine.

### Contrast discrimination—without adaptation

Contrast discrimination thresholds were very similar in migraine and control groups, increasing with increasing base contrast. The slope of the power function relating increment threshold to base contrast was approximately 0.6 in both groups. This lies within the range reported in the literature on contrast increment thresholds for studies employing grating or Gabor stimuli (see Legge [29] for review). We are aware of only one study of increment thresholds for stimuli with low spatial and high temporal frequencies. Anderson and Vingrys (38) employed small spots with hard edges (rather than Gaussian profiles used in our study), and square-wave flicker. Their data show similar slopes for 4- and 20-Hz flicker and the slopes, while not stated, appear [Figure 5 in ref. (38)] to be similar to those of the present study.

**Figure 6.**
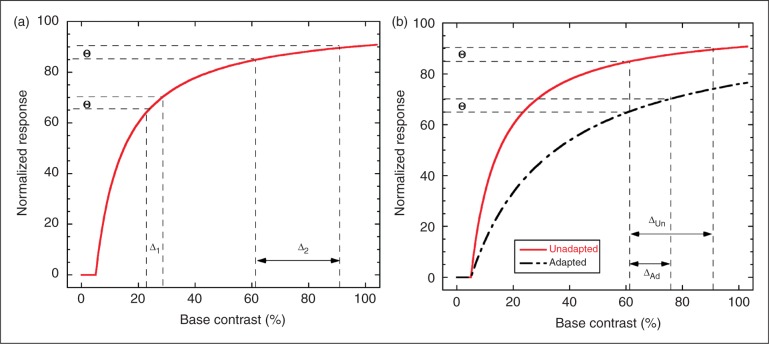
Role of compressive, saturating neural response non-linearity in generating increment thresholds. (a) Normalized neural response with threshold and saturating suprathreshold response is plotted. To achieve a criterion increment θ in the response requires a contrast stimulus increment of Δ1 at low base contrast (near 20% in plot) but a significantly larger stimulus increment, Δ2, at a high base contrast. This effect of contrast compression explains the observed increase of increment thresholds with base contrast. (b) Comparison of unadapted baseline response (solid line) with adaptation caused by divisive reduction of input stimulus (dashed line). At a base contrast near 60%, the unadapted increment threshold is ΔUn. Adaptation produces the much smaller increment threshold ΔAd, because adaptation keeps the response function further from saturation. This explains the crossing of adapted migraine increment thresholds at high contrast relative to the minimally adapted control increment thresholds.

Increment threshold curves are assumed to reflect non-linearities in the underlying neural stimulus-response function for individual neurons and/or for the ensemble of neurons forming the part of the visual pathway supporting the measured thresholds. As illustrated in Figure 6a, these stimulus-response functions typically show a compressive response with a steeper slope at low-to-mid contrasts, which flattens as the neurons approach saturation. Because of the steepness of the response curve at low contrast, a small stimulus increment Δ_1_ (shift along the x-axis) produces a change in response (Θ) large enough to be perceptible in a discrimination task. At the high end of the function, it requires a much larger increment in stimulus (Δ_2_) to produce a comparable (and hence detectable) change in response. This pattern yields the power law relationship linking threshold contrast increment to base contrast that is seen in Figure 4. At sufficiently high contrasts, the increment stimulus can be increased to 100% contrast without eliciting a sufficiently large response change to be detectable (saturation). Responses at the very low end of the function (below 5%) can be complex, involving a so-called “dipper function”, where the increment threshold dips below the absolute threshold (39). As we did not examine this region of the function systematically, our results should not be extrapolated to very low contrasts.

The fact that the slopes in the 10–70% contrast range were very similar for migraineurs and controls without adaptation, and there was no significant difference in baseline increment thresholds at 10% or 70% contrast, suggest that, under normal conditions of brief stimulation, the migraine visual system responds normally to temporal variation across a wide contrast range.

### Contrast discrimination thresholds—following adaptation

At suprathreshold contrasts we found a marked difference between migraineurs and controls in the slope of the increment threshold functions resulting in a crossover pattern for migraineurs but not controls. A similar pattern of adaptation involving slope change has been reported in two contrast discrimination studies in normal subjects that did not involve flicker (40,41). However, other studies of contrast discrimination in visually normal subjects have failed to find any effect of contrast adaptation over the range of base contrasts we have examined, despite the fact that both found clear elevations at contrast detection threshold, as reported here (42,43). Thus, our control participants show the same pattern of results reported by Määttänen and Koenderink (42) and by Ross et al. (43), whereas our migraineurs’ results are more similar to the findings of Greenlee and Heitiger (40) and Wilson and Humanski (41).

The implication of the crossover effect is that the neural response function has been flattened at lower contrasts (10%), and has become steeper at high contrast relative to the unadapted function (Figure 6b). Wilson and Humanski were able to model their crossover effect using feedback inhibition (41). For such a model to explain our data, stronger inhibition would be necessary in migraineurs than in controls. To date, the major claim concerning inhibition in migraine is that it is weaker, not stronger (44,45); however, a number of studies have failed to find support for any abnormality in inhibition strength (10,46–48). It should be noted that all of these studies used visual measures known to implicate cortical mechanisms (binocular interactions, orientation and spatial frequency tuning); however, the flicker adaptation effects under investigation here could well occur at an earlier stage of the visual pathway. Mechanisms other than feedback inhibition may be capable of producing similar changes in the slope of the increment threshold function. For example, a greater reduction in synaptic efficacy (synaptic depression) in migraineurs than in controls would effectively divide the input, thus resulting in increased slope at higher contrasts by postponing saturation. This would produce the crossover effect reported here.

### Habituation, adaptation and gain control

It has been claimed that migraineurs show abnormal habituation in the face of repetitive stimulation and may in fact show “potentiation” (27,49). This pattern has been reported both in behavioural responses (blink reflex [50–52]) and in electrophysiological findings of several sorts (visual [21,49], auditory [22,53], somatosensory [25] and nociceptive [54] evoked potentials and contingent negative variation [55–57]). It is important to ask whether the adaptation effects we report here are related to the habituation findings in this literature, in particular to the reported potentiation of the VEP with repeated stimulation. One of the defining characteristics of habituation is that a weak stimulus will produce greater habituation than a very strong stimulus (58). In the data reported here, we used a very high contrast adaptor (70% contrast) in all conditions, so our main results do not speak to this issue. However, in pilot work carried out in planning this study, we did try lower adapting contrasts as well (10% contrast) and in all cases found adaptation effects (both threshold elevation and facilitation) to be smaller with weaker (lower contrast) adapting stimuli, which led to the choice of 70% contrast adaptors in the work reported here. It should be noted that the existing evoked potential studies do not address this criterion for habituation, as they always entail stimulation with very high contrast checkerboard stimuli. We intend to pursue this question more rigorously in future psychophysical work and hope that it will also be investigated electrophysiologically. In the meantime, we prefer the term “adaptation”, which has been widely used in the sensory literature to describe changes in both neural response and percept following prolonged stimulation.

Traditionally, adaptation was considered to reflect neural or synaptic “fatigue”; more recent work indicates that adaptation may involve more complex processes (59–61). One role for adaptation that has been extensively explored is gain control—the adjusting of the operating range of the system to match the prevailing stimulus conditions. The increase in sensitivity seen in migraineurs at 70% contrast, after adaptation to the same 70% stimulus, would be predicted by a gain control model; sensitivity is increased around the prevailing contrast level at the expense of low contrast sensitivity. It was once thought that contrast gain was a property only of cortical neurons (62,63) and that gain controls in the retina were concerned only with luminance gain. However, there is now convincing evidence in a range of mammals that contrast adaptation mechanisms are active in the retina (64–66) and are also evident in neural responses in both the lateral geniculate nucleus LGN (67) and in the pretectum (68). Whether these are entirely inherited in the retinal input or whether additional adaptive processes occur at these subcortical visual nuclei remains uncertain. Thus, the threshold differences between migraineurs and controls seen after adaptation in the present study may reflect retinal, subcortical or cortical differences, and it will require further studies employing both psychophysics and brain imaging approaches to unravel this problem. One important point to be emphasised is that there are likely at least two processes at play following adaptation. One elevates detection threshold but has little effect on discrimination thresholds at higher base contrasts. This process appears to be exerting comparable effects in migraineurs and in controls. However, a second process is also evident which acts in a divisive fashion to change the slope of the suprathreshold function; this process is significantly stronger in migraineurs than in controls. Developing a full model of these interacting processes is beyond the scope of the present study.

### Perceived contrast

An important unanswered question is whether the perceived contrast of our stimuli changed with adaptation. Reductions in perceived contrast were reported in normal subjects following adaptation even though increment thresholds were unaffected (42). In a study of static grating patterns, Shepherd reported reduced perceived contrast in migraineurs compared to controls for the highest contrast patterns within a set (7). On the other hand, the aversiveness experienced by migraineurs in the presence of prolonged flicker suggests that the flickering spots might be perceived to have even higher contrast after adaptation. However, it is important to recognize that perceived aversiveness and perceived contrast might be carried by different neural pathways. Recent findings strongly suggest the involvement of a subcortical pathway outside the geniculostriate system in mediating photophobia to bright light in migraine (69). The lack of correlation between discomfort scores and thresholds in the present study would be consistent with their mediation by different neural pathways. However, it must be emphasised that the discomfort scores were collected only once, whereas the threshold data were accumulated over multiple sessions, so inter-session variability could also account for the absence of significant correlations.

## Conclusions

The present results confirm that visual flicker at high temporal frequencies is not only aversive to individuals with migraine but also affects their visual systems differently in other measurable ways. While most of the vision research related to migraine has focused on cortical mechanisms, due in large part to the very strong evidence that visual auras originate cortically (70–72), we would suggest that the heightened sensitivity of migraineurs may lie at many levels of the visual pathway, possibly beginning as early as the retina, and that the stimuli that cause migraineurs most discomfort are those that are likely to give us greatest insight into the nature of the visual abnormalities in migraine.
